# Fitness benefits of the fruit fly *Rhagoletis alternata* on a non-native rose host

**DOI:** 10.1007/s00442-015-3524-y

**Published:** 2016-01-18

**Authors:** Kim Meijer, Christian Smit, Menno Schilthuizen, Leo W. Beukeboom

**Affiliations:** Groningen Institute of Evolutionary Life Sciences (GELIFES), University of Groningen, P.O. Box 11103, 9700 CC Groningen, The Netherlands; Altenburg & Wymenga, Ecological Consultants, P.O. Box 32, 9269 ZR Veenwouden, The Netherlands; Naturalis Biodiversity Center, Darwinweg 2, 2333 CR Leiden, The Netherlands

**Keywords:** Herbivory, Non-native species, Parasitization, Enemy escape, Fitness

## Abstract

Many species have been introduced worldwide into areas outside their natural range. Often these non-native species are introduced without their natural enemies, which sometimes leads to uncontrolled population growth. It is rarely reported that an introduced species provides a new resource for a native species. The rose hips of the Japanese rose, *Rosarugosa*, which has been introduced in large parts of Europe, are infested by the native monophagous tephritid fruit fly *Rhagoletis alternata*. We studied differences in fitness benefits between *R. alternata* larvae using *R. rugosa* as well as native *Rosa* species in the Netherlands. *R. alternata* pupae were larger and heavier when the larvae fed on rose hips of *R. rugosa*. Larvae feeding on *R. rugosa* were parasitized less frequently by parasitic wasps than were larvae feeding on native roses. The differences in parasitization are probably due to morphological differences between the native and non-native rose hips: the hypanthium of a *R. rugosa* hip is thicker and provides the larvae with the possibility to feed deeper into the hip, meaning that the parasitoids cannot reach them with their ovipositor and the larvae escape parasitization. Our study shows that native species switching to a novel non-native host can experience fitness benefits compared to the original native host.

## Introduction

Over the past centuries, many species of plants and animals have been introduced into new areas worldwide, both intentionally and unintentionally (Williamson 1996). Some of these species manage to establish, integrating into the novel ecosystems and interacting with the native species present. Such non-native species can have large negative effects on economics (Pimentel et al. 2005), human health (Ziska and Caulfield 2000) and native ecosystems (Williamson 1996). In many cases, non-native species have become very successful (i.e., invasive) and negatively affect native species by competition or predation (e.g. Pelicice and Agostinho 2008; Perdereau et al. 2010). However, conversely, non-native species may also provide new niches for native species to utilize, which ultimately may lead to population differentiation and the evolution of new host races or (sub)species.

The best described example of such a host shift comes from the North American apple maggot fly *Rhagoletis pomonella* (Diptera: Tephritidae). This species shifted from the native hawthorn (*Crataegus* spp.) to introduced apple (*Malus domesticus*) (Bush 1969; McPheron et al. 1988). Within 400 years, the populations on both hosts became genetically differentiated host races (Feder et al. 1988; McPheron et al. 1988), differing in behavior, host preference and timing of reproduction (Feder and Filchak 1999; Filchak et al. 2000; Prokopy et al. 1988). Similar examples of a shift to a non-native host plant have been documented in, e.g., the goldenrod gall midge *Dasineura folliculi* (Diptera: Cecidomyiidae) (Dorchin et al. 2009), the European corn borer *Ostrinia nubilalis* (Lepidoptera: Crambidae) (Bethenod et al. 2005; Thomas et al. 2003) and the soapberry bug *Jadera haematoloma* (Hemiptera: Rhopalidae) (Carroll and Boyd 1992). These host shifts are often accompanied by morphological, physiological and behavioral changes in the herbivores.

Even though non-native plant species may be colonized by native herbivores, they are in general attacked less than native species (Colautti et al. 2004; Meijer et al., unpublished). This escape from enemies is most likely one of the factors influencing the success of non-native plants (and other organisms), as predicted by the Enemy Release Hypothesis (Williamson 1996). Interestingly, native herbivore species that (partly) shift to a non-native host species may in turn benefit from escape of their native enemies, if predators and parasites are less likely to visit the non-native plant. For example, Feder (1995) showed that *R. pomonella* larvae are parasitized much less on the non-native *Malus domesticus* (13 %) than on the native host plant *Crataegus* species (46 %). Very few native/non-native systems have been studied in detail in terms of such tri-trophic interactions. Such studies are, however, needed to understand and predict the success of non-native species. In this study, we focus on the three-way interaction between plants, their herbivorous (phytophagous) insects and the parasitoids of the herbivores. In the Netherlands, larvae of the tephritid fruit fly *R. alternata* (Diptera: Tephritidae) feed on the fruits of native rose species (*Rosa* spp.) and the non-native Japanese rose (*Rosa rugosa*). The larvae are parasitized by several parasitic wasp species (Hymenoptera: Braconidae). We test whether there are differences in larval size, parasitization frequency and accessibility by parasitic wasps between larvae feeding on the fruits of native and non-native roses.

## Methods

### Study species

The larvae of the European rose-hip fruit fly, *R. alternata* (Diptera, Tephritidae), are monophagous fruit herbivores of rose hips. Nowadays, the most common hosts in Europe are native species of the *Rosa canina* complex as well as the introduced Japanese rose *R. rugosa* (Leclaire and Brandl 1994). *R. alternata* is univoltine (one generation per year) with adults emerging in early June. Eggs are laid under the skin of rose hips from June until August. The larvae feed in the hypanthium of the fruit until October, after which the mature third-instar larvae leave the fruit to pupate in the soil (Bauer 1986). *R. rugosa* is native in Japan, Kamchatka and northeastern China (Weidema 2006). It was first recorded in Europe in 1796, but has now been reported from 15 different countries in Western, Central and Eastern Europe. It is cultivated in parks, gardens and along roads, and it has also become established in many nature areas (Leclaire and Brandl 1994). It flowers somewhat earlier than the native *R. canina*; hips ripen at the turn of August to September, whereas fruits of the native roses ripen in October. Hip densities are equal between native roses and *R. rugosa*, but the hips of *R. rugosa* are larger than those of native roses. Therefore, hip biomass per unit bush area is higher in *R. rugosa* (Leclaire and Brandl 1994). Parasitic wasps are the main enemies of *Rhagoletis* larvae (Bauer 1986). *Scambus annulatus* (Hymenoptera: Ichneumonidae), *Utetes magnus* (synonym *Opius magnus*) and *Psyttalia carinata* (synonyms: *P. rhagoleticola*, *Opius rhagoleticola* and *O. carinata*) (Hymenoptera: Braconidae) have been reported as parasitoids of *R. alternata* (Bauer 1986; Hoffmeister 1992).

### Collection

In September and October 2010, rose hips were collected on three different locations in the Netherlands: Haren, province of Groningen (53.17°N 06.61°E), Ameland, province of Friesland (53.45°N 05.77°E) and Schiermonnikoog, province of Friesland (53.49°N 06.22°E). On each location, both native roses (*Rosa* spp.) and non-native roses (*R. rugosa*) were sampled in an area of 50–100 km^2^. Approximately 20–50 rose hips per plant were collected from a total of 50 plants in these three areas together (24 native and 26 non-native roses). The rose hips were stored in containers (l × w × h: 16.5 × 12 × 6 cm) and covered with fine netting (mesh size <1 mm). The containers were kept outdoors, protected from rain and direct sunlight to mimic natural weather conditions. Within a few days after collection, *R. alternata* larvae emerged from the rose hips, pupating soon after. Pupae were collected and stored individually in tubes (h × ø: 6.5 × 1 cm) under these same outdoor conditions. The next spring the containers were checked for emerging adults several times per week. In the summer all remaining pupae were checked once more and scored into three categories: either an adult fly or adult parasitoid wasp had emerged, or the pupa had died during the winter.

### Pupal size, weight and parasitization rate

About three weeks after collection, the size (length, measured under a binocular microscope) and weight of the pupae were measured (accuracy 0.05 mm and 1 μg, resp.). As size and weight of the pupae were highly correlated (*R*^2^ = 0.759), only size was measured for all pupae and weight (which was much more labor intensive) for only a sub-sample. The measurements on the pupae from which flies emerged were used to test for differences between individuals feeding on the native and non-native rose, using a linear mixed model. The model included status (native vs. non-native rose) as a fixed factor and plant individual and collection location as random factors. The difference in parasitization rate (number of wasps/total number of pupae that yielded flies or wasps) between native and non-native roses was tested using a generalized linear mixed model (GLMM) with binomial error terms, again with status as a fixed factor and plant individual and collection location as random factors.

### Accessibility of larvae by parasitic wasps

**Fig. 1:**
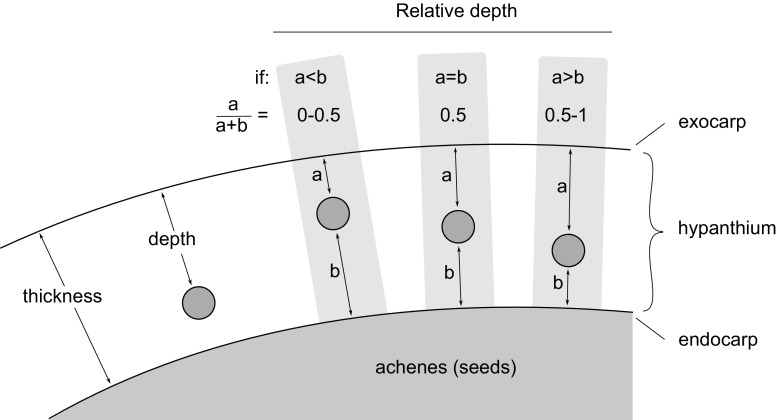
Thickness measurement of the outer layer of rose hips (hypanthium). Only this layer is edible for *Rhagoletis alternata* larvae. The thickness of this layer was measured from *top* (exocarp) to *bottom* (endocarp). The depth of the larval mines in the hypanthium was measured from the exocarp to the top of the mine. The relative depth of the larval mines was calculated by: *a*/*a* + *b*, resulting in a ratio from 0 to 1 (*a* the distance between the exocarp to the *top* of the mine; *b* the distance between the endocarp to the *bottom* of the mine)

**Fig. 2:**
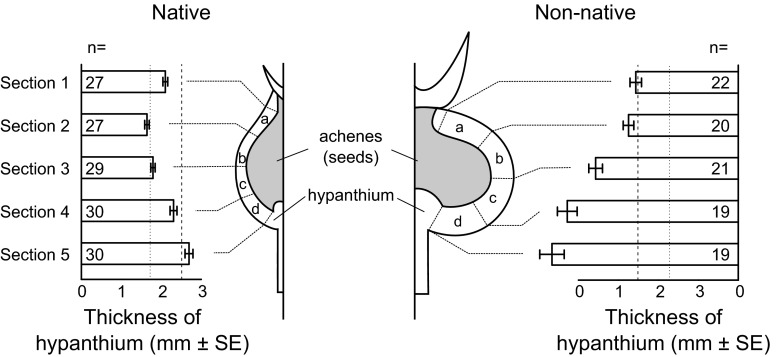
Thickness of rose hip hypanthium in relation to parasitic wasp ovipositor length. Fruit fly larvae can escape parasitization if they live deep enough in the hypanthium. Shown are cross sections of a native (*left*, *Rosa* sp.) and non-native (*right*, *R. rugosa*) rose hip. Thickness of the hypanthium was measured at five different positions, ranging from the *top* to the *bottom* part of the rose hip. *Bars* show the average thickness of the rose hips, including standard errors and sample sizes. The depth of the larval mines was measured at four different sections of the hypanthium (*a*–*d*) (data not shown in this figure). The *vertical lines* represent the average length of the ovipositor of the parasitic wasps; *dotted line*: *Utetes ferrugator*, *dashed line*: *Psyttalia carinata*

To determine the accessibility of the fly larvae by the parasitic wasps, the length of the ovipositor of the emerged parasitic wasps was compared to the thickness of the hypanthium, and to the depth of the mines made by the *R. alternata* larvae for both the native and non-native roses. The length of the ovipositor was measured under a binocular microscope (accuracy 0.05 mm). To determine the thickness of the hypanthium and the depth of the mines, digital photos were made of cross sections of hips (collected in autumn 2011), accompanied by a ruler. Inkscape 0.48 (inkscape.org) was used to measure the thickness of the hypanthium and the mine depth (Fig. 1). The thickness of the hypanthium was measured at five positions, ranging from the top to the bottom of the rose hip (Fig. 2). The depth of the larval mines was measured in four sections, ranging from the top to the bottom (indicated by a–d in Fig. 2). The distance was measured between the exocarp and the larval mine (a), and between the endocarp and the larval mine (b). Subsequently, the relative depth of the larval mines was determined as: *a*/(*a* + *b*), ranging from 0 [touching the exocarp (outer layer)] to 1 (touching the endocarp/seeds) (Fig. 1).

Both the differences in thickness of the hypanthium and depth of the larval mine between native and non-native roses were tested using a linear mixed model, with status (native vs. non-native) as a fixed factor, and rose hip individual and measuring position/section as random factors. The difference in thickness of the hypanthium was tested separately for all five positions, using a linear mixed model with status as fixed factor and rose hip individual as random factor. The depth of the larval mines was tested for all four separate sections, using a linear mixed model with status as fixed factor and rose hip individual as random factor. If larvae have no preference for feeding either deep or shallow, the average relative depth will be 0.5. If, on the other hand, larvae do prefer to feed either deep or shallow, the average relative depth will be, respectively, higher or lower than 0.5. We tested whether the relative depth of larval mines was different between native and non-native rose hips. Furthermore, we tested if the relative depth of the larval mines was equal, higher or lower than 0.5.

All analyses were done in R (R Development Core Team 2010), using the lme4 library (Bates et al. 2011). In all analyses, the effect of the fixed factors was tested by comparing the model with and without this factor, using ANOVA.

## Results

A total of 1366 *R. alternata* pupae were collected, 1078 from native and 288 from non-native rose hips. Three-hundred-and-twenty pupae (23.43 %) died during winter. From the remaining 1046 pupae, 953 (69.77 %) adult *R. alternata* flies and 93 (6.81 %) parasitic wasps emerged.

### Size and weight

**Fig. 3:**
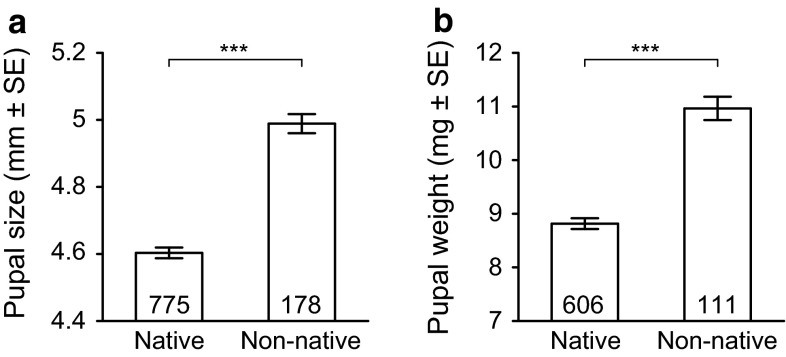
Size (**a**) and weight (**b**) of pupae of *Rhagoletis alternata* that emerged from native roses (*Rosa* spp.) and non-native roses (*R. rugosa*). Shown are the average ±SE, including sample size and significance level (***p < 0.001). *Note* Y-axis is truncated

Pupae collected from non-native rose hips were significantly larger (7.14 %) and heavier (22.79 %) than those from native rose hips (Fig. 3; Table 1). Size and weight of the pupae were highly correlated (*R*^2^ = 0.759, Table 1).

**Table 1:** Overview of the statistical analysis of the effect of host (native vs. non-native) on herbivore size, weight, and parasitization rate, the thickness of the rose hip hypanthium and the absolute and relative depth of the mines

**Size and weight**	*n*	*χ* ^2^	*df*	*p*
*Pupal size*—mixed model, random effects: *plant indiv.* and *coll. location*				
Fixed factor: s*tatus*	953	17.03	1	<0.0001
*Pupal weight*—mixed model, random effects: *plant indiv.* and *coll. location*				
Fixed factor: s*tatus*	717	9.27	1	0.0023

Correlation pupal size and weight	*n*	*t*	*df*	*p*
*R* ^2^ = 0.759	717	58.34	715	<0.0001

**Parasitization**	*n*	*Z*	*df*	*p*
*Parasitization rate*—GLMM, random effects: *plant indiv.* and *coll. location*				
Fixed factor: s*tatus*	1046	−2.41	1	0.0161

**Thickness of the mesocarp**	*n*	*χ* ^2^	*df*	*p*
*All positions*—mixed model, random effects: *rose hip indiv.* and *measuring position*				
Fixed factor: s*tatus*	244	56.83	1	<0.0001
*Positions separately*—mixed model, random effects: *rose hip indiv.*				
Position 1, fixed factor: *status*	49	11.15	1	0.0008
Position 2, fixed factor: *status*	47	45.00	1	<0.0001
Position 3, fixed factor: *status*	50	62.11	1	<0.0001
Position 4, fixed factor: *status*	49	42.58	1	<0.0001
Position 5, fixed factor: *status*	47	45.00	1	<0.0001

**Depth of the larval mines**	*n*	*χ* ^2^	*df*	*p*
*All selections*—mixed model, random effects: *rose hip indiv.* and *measuring section*				
Fixed factor: s*tatus*	166	12.61	1	0.0004
*Positions separately*—mixed model, random effects: *rose hip indiv.*				
Position a, fixed factor: *status*	46	1.82	1	0.1772
Position b, fixed factor: *status*	25	3.67	1	0.0554
Position c, fixed factor: *status*	49	0.81	1	0.3692
Position d, fixed factor: *status*	46	11.70	1	0.0007

**Relative depth of the larval mines**	*n*	*χ* ^2^	*df*	*p*
*All selections*—mixed model, random effects: *rose hip indiv.*				
Fixed factor: s*tatus × thickness hypanthium*	157	1.13	1	0.2883
Fixed factor: s*tatus*	157	1.63	1	0.2013
Fixed factor: *thickness hypanthium*	157	3.83	1	0.0504
*Overall depth*—one sample proportion test				
Deviation from 0.5	155	43.38	1	<0.0001

The thickness of the hypanthium was measured at five different positions, ranging from the top (position 1) to the bottom (position 5) of the rose hip. The absolute depth of the mines in the hypanthium was measured at four different sections in between these five positions (see Fig. 2 for details)

### Parasitization rate

**Fig. 4:**
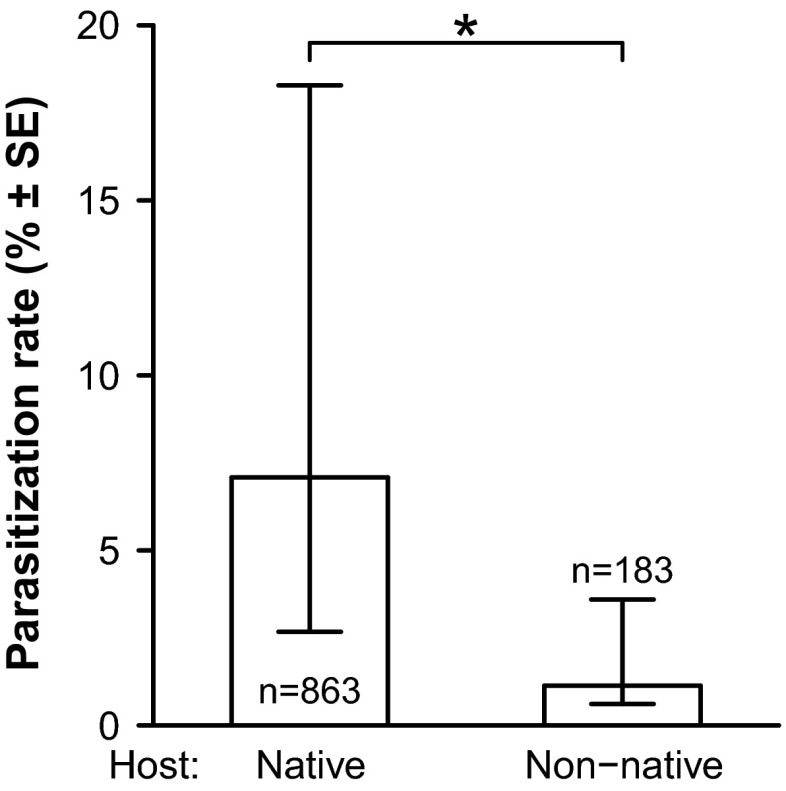
Parasitization rate of pupae from native roses (*Rosa* spp.) and non-native roses (*R. rugosa*). Shown are the average ±SE, sample size and significance level (*p < 0.05). Averages and SEs are derived from the model estimates that were back transformed using the inverse logit (average: exp(*p*)/(exp(*p*) + 1); SE: exp(*p* ± SE)/(exp(*p* ± SE) + 1))

Ninety-three of 1046 (8.89 %) *R. alternata* pupae were parasitized by the braconid wasps *U. ferrugator* (Goureau, 1862) and *Psyttalia carinata* (Thomson, 1895). *U. ferrugator* was by far the most common (87 individuals, 8.32 % of the fly pupae), whereas *P. carinata* was found only occasionally (six individuals, 0.57 % of the fly pupae). In both species, the sex ratio was female biased (31 and 33 % males, respectively). The parasitization rate of pupae collected on the native rose was significantly higher (almost four times) than on the non-native rose (Fig. 4; Table 1).

### Accessibility of larvae by parasitic wasps

**Fig. 5:**
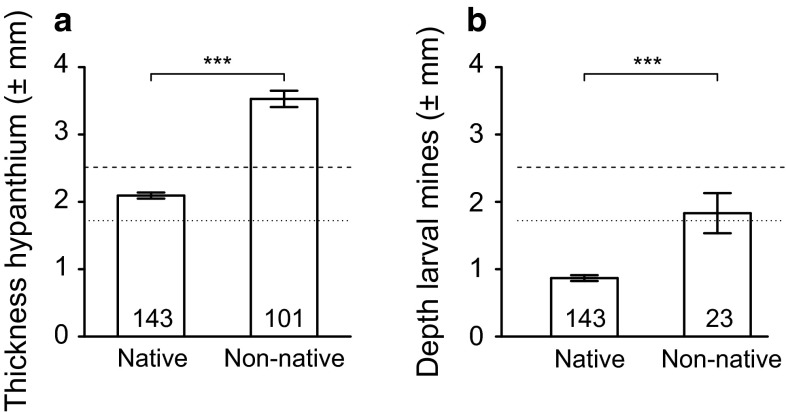
Thickness of the hypanthium (**a**) and depth of the larval mines (**b**) in native roses (*Rosa* spp.) and non-native roses (*R. rugosa*). Shown are the average ±SE, including sample size and significance level. Measurements of the thickness of the hypanthium at all five positions (Fig. 2, 1–5) and measurements of the depth of the larval mines at all four sections (Fig. 2a–d) are combined. The horizontal lines represent the average length of the ovipositor of the parasitic wasps; *dotted line*: *Utetes ferrugator*, *dashed line*: *Psyttalia carinata*

The hypanthium of the non-native rose hips was thicker than those of the native rose hips at all five positions measured (Table 1; Fig. 2). Overall, the hypanthium of the non-native rose hips was almost 70 % thicker than that of the native rose hips (Table 1; Fig. 5a). The ovipositors of both parasitic wasp species are short, 1.73 ± 0.02 mm (*n* = 58) in *U. ferrugator* and 2.51 ± 0.02 mm (*n* = 3) in *P. carinata*. The thickness of the hypanthium of the native rose hips was only slightly greater than the average length of the ovipositor of *U. ferrugator*, while in the non-native rose hips, the hypanthium was more than twice as thick as the length of the ovipositor (Fig. 5a, dotted line). However, the thickness of the hypanthium of the native rose hips was less than the average length of the ovipositor of *P. carinata*, and in non-native rose hips, it was only 40.6 % larger than the length of the ovipositor (Fig. 5a, dashed line).

**Fig. 6:**
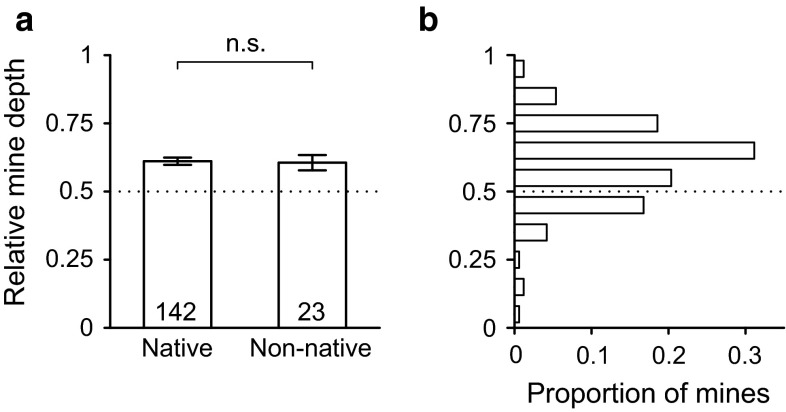
Relative depth of the larval mines in the hypanthium. The average relative depth of the larval mines in native and non-native rose hips (**a**); shown are the average ±SE, including sample size and significance level. The histogram shows the distribution of the relative depth of larval mines in native and non-native rose hips combined (**b**). In both graphs, the *dotted line* represents a relative depth of 0.5

Overall, the larval mines were more than twice as deep in the non-native rose hips compared to in the native rose hips (Table 1; Fig. 5b). The depth differed between native and non-native rose hips in two out of four sections (Table 1; Fig. 2). The average depth of the mines in the native rose hips was much less than the average length of the ovipositor of the wasps (Fig. 5b). Respectively, 95.8 and 99.3 % of the mines were within reach of the ovipositors of *U. ferrugator* and *P. carinata*. In the non-native rose hips, many larval mines were positioned deeper than the length of the ovipositor of the wasps, and only 65.2 and 82.6 % of the mines were within reach of the ovipositors of *U. ferrugator* and *P. carinata*, respectively. The relative depths of the larval mines did not differ between native and non-native rose hips (Table 1; Fig. 6a). Most larval mines (76.4 %) were in the deeper parts of the hypanthium, and in both the native and non-native rose hips, the average relative depth was larger than 0.5 (Table 1), i.e., on average the larval mines are in the inner part of the hypanthium, away from the access points of the wasps (Fig. 6b).

## Discussion

In this study, we compared the differences in size and parasitization frequency between larvae of the tephritid fruit fly (*R. alternata*) feeding on the fruits of native rose species (*Rosa* spp.) and the non-native rose *R. rugosa*. These differences were linked to the size of the rose hips and the accessibility of the fruit fly larvae by parasitoids. Differences in these fruit fly traits indicate possible fitness benefits for *R. alternata*.

The hips of *R. rugosa* are much heavier than the hips of native roses (Leclaire and Brandl 1994). Therefore, differences can be expected between herbivores that feed on them. Leclaire and Brandl (1994) showed that *R. alternata* larvae feeding on rose hips of *R. rugosa* are heavier and that their developmental time is shorter compared with larvae feeding on native roses. This corresponds with our findings that *R. alternata* pupae are both larger and heavier when the larvae fed on rose hips of *R. rugosa*. This size difference will probably give the flies a fitness advantage during their adult life, since larval body size is positively correlated with fecundity in many insect species (Liedo et al. 1992; Leclaire and Brandl 1994; Yoshimura 2003). On the other hand, fitness of flies and parasitoids may be differentially affected by the feeding of birds and other frugivores on the rose hips. If we assume that non-native hosts are eaten more often, this would result in an overestimation of fly and parasitoid survival on non-native roses. To our knowledge, no studies have thus far reported on differential rose hip consumption between native and non-native roses.

The difference in parasitization rate between the native and non-native roses was very large. *R. alternata* larvae were parasitized five times less frequently in *R. rugosa* hips than in the native roses. This means that larvae have increased survival chances when feeding on *R. rugosa*. Comparison of the length of the ovipositor of the wasps, the thickness of the hypanthium and the (relative) depth of the larval mines suggest that the larvae escape parasitization. In the hips of the native roses, most of the larval mines were within reach of the wasp’s ovipositor, while in the hips of the non-native roses, many were out of reach. Furthermore, the fact that most mines were positioned in the inner part of the hypanthium, away from the access points of the wasps, suggests active avoidance of parasitization by the larvae. Similar results have been found in the olive fruit fly *Bactrocera oleae* (Diptera: Tephritidae), which is parasitized by different species of braconid wasps, e.g., *Psyttalia concolor* and *Bracon celer* (Sime et al. 2006, 2007; Daane et al. 2008). The parasitization rate is affected by the thickness of the mesoderm of the olives (*Olea europaea*), ranging from ±60 % in small olives to <10 % in large olives (López et al. 1999; Wang et al. 2009a, b).

Parasitization rate may depend on several factors. Higher host densities may lead to higher parasitization rates because parasitoids stay longer in rich areas (Godfray 1994). We did not determine rose hip infestation rates, but Leclaire and Brandl (1994) found a threefold higher number of eggs per rose hip in a European study. We collected fewer non-native rose hips and found fewer larvae in them, but we do not know the number of larvae per rose hip. We can therefore not determine whether non-native roses provide a richer host environment for the parasitoids. However, we find a lower parasitization rate at non-native roses, which indicates that larvae in non-native rose hips have a lower chance of parasitization. There are a few aspects of our study that need further investigation. Determining actual parasitization rates requires the counting of eggs and measuring death of non-parasitized (flies) and parasitized hosts (wasps) at early developmental stages. Survival rates of fly eggs and larvae, either parasitized or not, might be influenced by pathogens in the rose hip. Although laboratory studies in Drosophila do not show severe effects on larval survival as a consequence of ovipositor intrusion, little is known about this under natural conditions. There may also be effects of phenology. The ripening of the non-native fruits is spread out over a larger time period. Depending on voltinism, early season larvae may experience a different parasitism rate than late season larvae. It is not exactly known at what stage of fruit development the flies oviposit and at what stage fly larvae are parasitized by the wasps. This may affect how strongly parasitization risk is dependent on ovipositor length and larval mine depth. Solving these issues requires more detailed studies of fly and wasp oviposition behavior in relation to the full rose ripening season.

Host shifting can lead to host-associated differentiation and speciation. This process has been documented especially frequently for herbivorous insects. Until now, no genetic differentiation has been found between *R. alternata* flies infesting different native roses (Kohnen et al. 2009; Vaupel et al. 2007), but it remains unknown whether there is host-associated differentiation between the populations on the native and those on the non-native roses. It is also of interest that host-associated differentiation in herbivorous insects can lead to host-associated differentiation in their parasites and predators (Stireman et al. 2006). The gall-inducing fly *Eurosta solidaginis* (Diptera: Tephritidae), for example, formed two different host races on *Solidago altissima* and *S. gigantea. E. solidaginis* is preyed upon by the tumbling flower beetle *Mordellistena convicta* (Coleoptera: Mordellidae). The predator *M. convicta* in turn developed host races that differ in emergence time, mate assortatively, prefer flies on the natal host plant, and have higher survival on flies from native host plants (Eubanks et al. 2003). Similarly, the parasitic wasp *Diachasma alloeum* (Hymenoptera: Braconidae) diverged into two incipient species following the divergence of its host, apple maggot fly, *Rhagoletis pomonella*. The native hosts are hawthorns (*Crataegus* spp.) and the non-native hosts are apples (*Malus domesticus*) (Forbes et al. 2009).

Our results contribute to the understanding of what can happen to non-native species that are introduced into new areas. Many such introductions may go unnoticed because the introduced species is not adapted to its new environment and dies out. In some cases, however, species may become established and rapidly increase in number, acquiring the status of a pest. Such species often have few or no natural enemies in their novel environment. The system we studied, however, consists of an introduced plant being used as host by a native herbivore. We found clear fitness advantages of *R. alternata* larvae feeding on non-native rose hips compared to native rose hips. We do not know whether the establishment or competitive ability of *R. rugosa* is affected by this herbivory, but our data show that introduction of a novel plant can create a new niche for a native insect. Moreover, they reveal that a native herbivore that switches to a novel non-native host can experience fitness benefits through release from parasitization compared to its original native host. Whether this in turn increases the competitive ability of the native roses requires further investigation.
